# Response of net primary productivity to grassland phenological changes in Xinjiang, China

**DOI:** 10.7717/peerj.10650

**Published:** 2021-04-30

**Authors:** Renping Zhang, Jing Guo, Gang Yin

**Affiliations:** 1 College of Resource and Environment Sciences, Key Laboratory of Oasis Ecology, Xinjiang University, Urumqi, China; 2 Xinjiang Academy Forestry, Urumqi, China

**Keywords:** Vegetation, Net primary productivity, Phenology, Xinjiang, Correlation

## Abstract

Determining the relationship between net primary productivity (NPP) and grassland phenology is important for an in-depth understanding of the impact of climate change on ecosystems. In this study, the NPP of grassland in Xinjiang, China, was simulated using the Carnegie-Ames-Stanford approach (CASA) model with Moderate Resolution Imaging Spectroradiometer (MODIS) grassland phenological (MCD12Q2) data to study trends in phenological metrics, grassland NPP, and the relations between these factors from 2001–2014. The results revealed advancement of the start of the growing season (SOS) for grassland in most regions (55.2%) in Xinjiang. The percentage of grassland area in which the end of the growing season (EOS) was delayed (50.9%) was generally the same as that in which the EOS was advanced (49.1%). The percentage of grassland area with an increase in the length of the growing season (LOS) for the grassland area (54.6%) was greater than that with a decrease in the LOS (45.4%). The percentage of grassland area with an increase in NPP (61.6%) was greater than that with a decrease in NPP (38.4%). Warmer regions featured an earlier SOS and a later EOS and thus a longer LOS. Regions with higher precipitation exhibited a later SOS and an earlier EOS and thus a shorter LOS. In most regions, the SOS was earlier, and spring NPP was higher. A linear statistical analysis showed that at various humidity (*K*) levels, grassland NPP in all regions initially increased but then decreased with increasing LOS. At higher levels of *K*, when NPP gradually increased, the LOS gradually decreased.

## Introduction

The Fifth Assessment Report by the Intergovernmental Panel on Climate Change noted that the average surface temperature of Earth increased by 0.85 °C from 1880–2012 and that temperatures were higher during the three consecutive decades between 1983 and 2012 than during any previous decades since 1850 (Qin & Stocker, 2014). In particular, temperature increases have been more pronounced at high latitudes (Fang et al., 2013; Guli et al., 2015). Climate change will affect vegetation phenology (Tateishi & Ebata, 2004) and lead to changes in vegetative productivity and structural composition as well as exchanges of water, heat, and carbon between soils, plants, and the atmosphere, which will in turn affect the climatic system and accelerate global warming (Monson et al., 2005; Wipf, Stoeckli & Bebi, 2009). Vegetation phenology is an important indicator of environmental conditions and climate change (Jeong et al., 2011). Vegetation productivity is an important factor that maintains the healthy and stable operation of an ecosystem (Melillo et al., 1993). Therefore, studying the relationship between vegetation phenology and productivity with respect to climate change is very important for understanding the impacts of phenological changes on ecosystem dynamics.

Studies on the influence of phenological changes on vegetation productivity have demonstrated that vegetation phenology is closely related to the net primary productivity (NPP) of ecosystems (Kimball et al., 2006; Piao et al., 2008; Richardson et al., 2010; Wang et al., 2017; Still et al., 2003). The start of the growing season (SOS) of vegetation plays a decisive role in its productivity level (Botta et al., 2010; Richardson et al., 2010). Research has also demonstrated that the end of the growing season (EOS) exerts an observable impact on vegetation productivity (Piao et al., 2008). Changes in the phenological period not only affect the growth process and degree of vegetation cover but also significantly affect vegetative productivity (Wang et al., 2017). An increase in temperature will prolong the growing season for vegetation but will also affect its metabolic processes (e.g., photosynthesis, respiration, and transpiration). Additionally, an increase in temperature will increase the consumption of organic matter by vegetation (Wipf & Rixen, 2010; Dragoni et al., 2011). Research has shown that climate change will prolong the growing seasons of vegetation in terrestrial ecosystems. For example, Keenan et al. (2014) studied the relationship between phenological period and carbon budgets of terrestrial ecosystems by using long-term phenological observations, fixed-point carbon dioxide (CO_2_) flux monitoring, and model simulations. The authors found that a prolonged growing season could help increase the net carbon uptake of an ecosystem. However, Elmore, Nelson & Craine (2016) demonstrated that a prolonged growing season might lead to a decrease in vegetation NPP. High temperatures increase the length of the growing season (LOS) and vegetative respiration. Therefore, the ways in which vegetation NPP varies require further investigation.

Xinjiang is located in the northwestern arid-semiarid region of China. With a vast land area, complex natural environmental conditions, considerable regional variation in hydrothermal resources, and diverse vegetation communities, Xinjiang provides a favorable research area for studying the correlations between vegetation dynamics and climate change at various time scales (Du et al., 2017). In recent decades, the average, daytime, nighttime, and extreme temperatures in Xinjiang have increased considerably. Additionally, precipitation and evaporation in Xinjiang have increased in a fluctuating manner (Fang et al., 2013; Wang et al., 2013). These changes in climatic conditions have caused substantial impacts to vegetation processes in Xinjiang. Since the 1980s, with the transition from a warm and dry climate to a warm and wet climate, there has been a considerable increase in overall vegetation greenness (Guli et al., 2015; Du et al., 2017) and an increase in the NPP of the vegetation in Xinjiang (Zhang et al., 2018). While the response of vegetation to climate change has been investigated (Du et al., 2017; Zhang et al., 2018), studies on the relationship between vegetation phenology and productivity are scarce.

Since it was proposed by Potter & Klooster (1997) and Field, Randerson & Malmström (1995), the Carnegie-Ames-Stanford approach (CASA) model has been shown to be capable of effectively estimating the vegetation NPP over a large area. The parameters of the CASA model vary by location and time, and they are corrected based on corresponding environmental conditions (Crabtree et al., 2009; Zhou et al., 2015; Zhang et al., 2016, 2018). Currently, the data used for studying phenological changes originate from two primary sources, namely, conventional ground-based phenological observations and remote sensing-based phenological observations. The ground-based phenological observations and related research methods are accurate and objective. However, except in certain developed countries in Europe and North America, data based on global ground-based phenological observations are extremely limited and insufficient to facilitate long-time series, broad-scale, and spatially continuous phenological analyses (Cong et al., 2013). In comparison, remote sensing data can reflect the spatially continuous phenological characteristics of vegetation over large areas and long periods of time (Yu et al., 2003). Zhang et al. (2003) fitted Moderate Resolution Imaging Spectroradiometer (MODIS)-enhanced vegetation index time series data from the northeastern U.S. to a logistic function. This approach does not require a defined phenological threshold for vegetation and is thus applicable to phenological monitoring over large areas. Based on this approach, the U.S. National Aeronautics and Space Administration generated a phenological product (MCD12Q2) that has been used extensively to study seasonal phenology and interannual variation across the global surface (Zhang et al., 2013; Xiao et al., 2013; Begue et al., 2014).

In this study, we investigated the temporal trends and spatial variability of grassland phenology and NPP dynamics and examined the mechanisms underlying the responses of NPP to phenological shifts in Xinjiang during 2001–2014. This study focused primarily on the following key issues: (1) the spatial distribution of and variation in grassland phenological metrics and NPP of the study area, (2) the relationships between phenological metrics and NPP, and (3) the effects of climatic factors on the NPP and phenological metrics of the grassland. Elucidating the above issues will help improve our understanding of the relations between grassland phenological metrics and NPP and thereby provide a scientific basis for sustainable ecosystem development in arid-semiarid regions.

## Materials and Methods

### Study area

**Figure 1:**
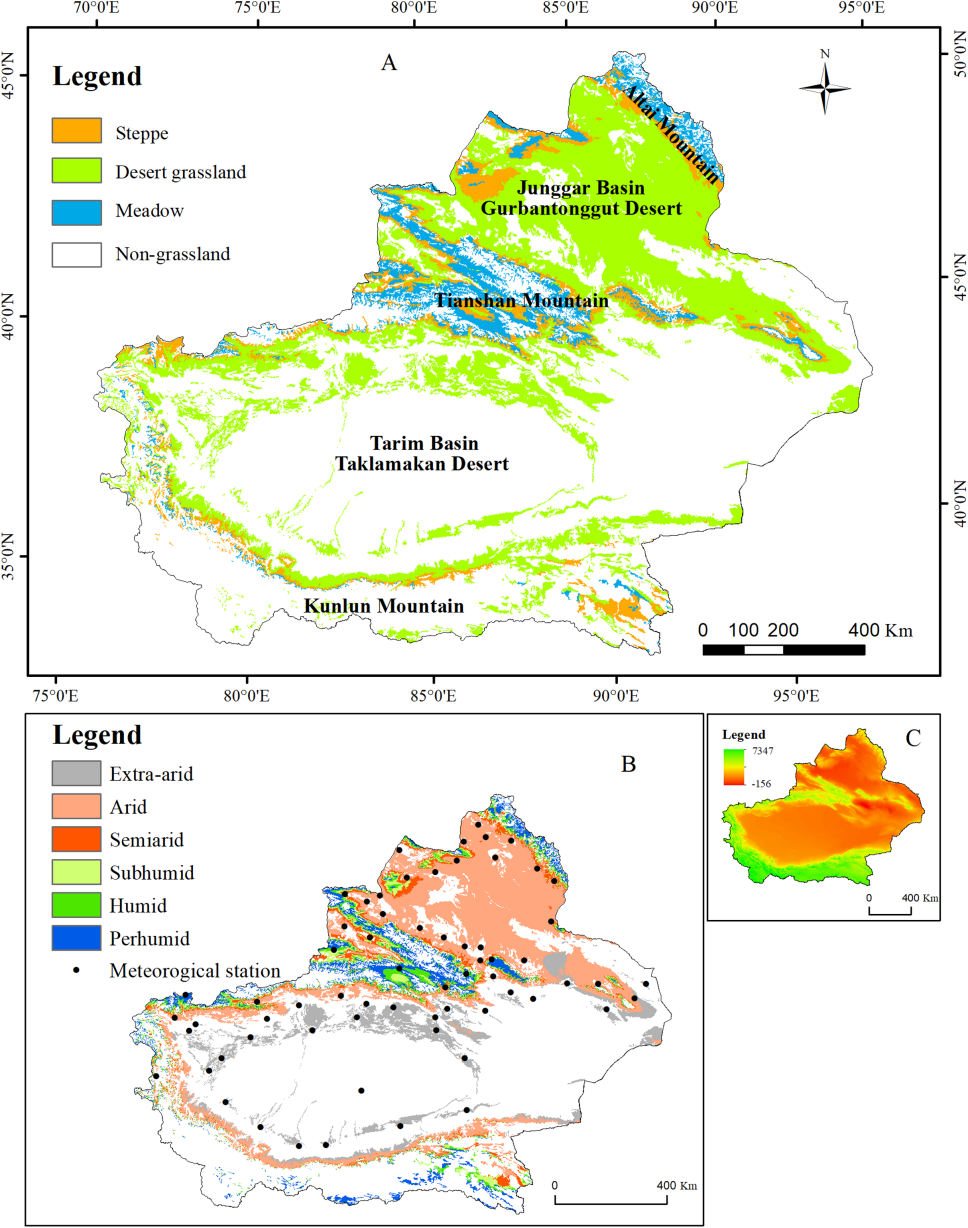
Geographic location of the study area. (A) The distribution of the grassland types in Xinjiang; (B) the distribution of humidity and meteorological stations in Xinjiang; (C) the distribution of elevation in Xinjiang.

The Xinjiang Uyghur autonomous region (N 34°22′–49°33′, E 73°22′–96°21′) is located in northwestern China and has an area of 166 × 104 km^2^, accounting for approximately 1/6 of the country’s land area. This region is situated in the center of the Eurasian continent and is surrounded by high mountains, with the Altai Mountains in the north, the Kunlun Mountains in the south, and the Tianshan Mountains in the central part. Two vast basins, the Junggar and Tarim Basins, are surrounded by these high mountains. The mountains in the study area differ greatly in their vertical zonality, and large areas of forest and grassland vegetation occur in the Tianshan, Altai, and Kunlun Mountains. Typical temperate desert vegetation is distributed across the Junggar and Tarim Basins. These three mountain ranges and two basins form the unique geographical environment of Xinjiang (Fig. 1A). The climate of Xinjiang features a temperate continental arid and semiarid climate with an annual sunshine duration of 2,550–3,500 h, an average annual temperature of 9–12 °C, and a frost period of up to 180–220 d. The annual precipitation ranges from 100 to 200 mm in the north and is less than 100 mm in the south, whereas the annual potential evaporation ranges from 1,500 to 2,300 mm in the north and from 2,100 to 3,400 mm in the south. Because of the special geographic location, terrain conditions, and arid climate, few plant species and low vegetation coverage are observed; hence, the ecological environment of Xinjiang is fragile. Xinjiang represents the third largest grassland area in China, with approximately 57.26 million km^2^ of grassland, which accounts for 34.4% of the total area of Xinjiang. The grassland area accounts for 86% of the total vegetation area of Xinjiang and is 22 times as large as the forest areas in Xinjiang.

### Climate data

The meteorological data were obtained from the China meteorological science data-sharing service system (http://data.cma.cn/). The data include the monthly average temperature and total precipitation from 67 meteorological stations in Xinjiang from 2001 to 2014 (see Fig. 1B). Thin-plate smoothing spline interpolation was performed with ANUSPLIN to interpolate the meteorological data to produce raster images with 500-m spatial resolution (Plouffe, Robertson & Chandrapala, 2015).

### Grassland types

The 1:4,000,000-scale grassland type map was obtained from the Natural Resources Comprehensive Investigation Committee of the Chinese Academy of Sciences. A total of 11 grassland types in Xinjiang are grouped into three classes according to their properties: Desert grassland (Temperate desert, Temperate steppe-desert, and Alpine desert), Steppe grassland (Temperate meadow steppe, Temperate steppe, Temperate desert steppe, Alpine steppe, and Alpine desert steppe) and Meadow grassland (Mountain meadow, Alpine meadow and Lowland meadow) (Fig. 1B).

### Humidity (*K*)

*K* levels were classified according to regional biological and climatic characteristics (Liang et al., 2012). Specifically, thermal regions and *K* levels were combined to classify areas based on a quantified biological and climatic index using the 0 °C-based annual accumulated temperature (i.e., the number of 0 °C-based growing degree days (GDD0)) and *K* (Ren et al., 2008).

(1) K=MAP/0.1×GDD0

where *K* is humidity index, MAP is mean annual precipitation, 0.1 is the model adjustment factor, and GDD0 is the number of 0 °C-based growing degree days, which can be calculated using the following equation:

(2) $$GDD0=\overset{12}{\underset{i=1}{\sum }}func\frac{MT10max_{i}+MT10min_{i}}{2}-T_{b}\times MD10_{i}$$

where *MT*10*max*_*i*_ and *MT*10*min*_*i*_ are the average maximum and minimum temperatures in the *i*th month during the 10-year period, respectively; T_b_ is 0 °C; and *MD*10_*i*_ is the number of days in the *i*th month during the 10-year period. Xinjiang can be divided into six types of regions according to *K* levels: extra-arid, arid, semiarid, subhumid, humid, and perhumid.

### Phenological data

The MODIS (C5) vegetation phenological (MCD12Q2) data, which have a spatial resolution of 500 m for the period 2001–2014, were downloaded from NASA’s website (https://ladsweb.modaps.eosdis.nasa.gov/search/order/1/MCD12Q2). The MCD12Q2 product was generated using the 8-d enhanced vegetation index (EVI), which was calculated from nadir bidirectional reflectance distribution function-adjusted reflectance (NBAR) data. The MCD12Q2 product provides the SOS and EOS based on the rate of curvature change in the MODIS-EVI time series data. The LOS is the time interval between the EOS and SOS. We performed inversion of the phenology of grassland in Xinjiang using the phenological product MCD12Q2, and the verification accuracy was good (*R* = 0.76, *P* < 0.05, Bias = −2.84 d, root mean square error (RMSE) = 16.44 d) (Zhang et al., 2019). Therefore, the MCD12Q2 phenology data are suitable for estimating the SOS of the grassland in Xinjiang.

The MCD12Q2 product data were subjected to format conversion and projection transformation using MODIS Reprojection Tools software. Specifically, the hierarchical format of the MCD12Q2 product data was converted to the GeoTIFF format. The projection of the MCD12Q2 product data was transformed to World Geodetic System 1984 coordinates.

### NPP estimation using the CASA model

The remote sensing and climatic data-based CASA model is a light use efficiency model developed by Field, Randerson & Malmström (1995), and it has been extended to estimate NPP over a broad geographic scale (Potter & Klooster, 1997). The CASA model is determined by absorbed photosynthetically active radiation (APAR, MJ/m^2^) and light use efficiency ε (g C/MJ) and is described as follows:

(3) $$NPP_{\left(x,t\right)}=APAR_{\left(x,t\right)}\times \varepsilon_{\left(x,t\right)}$$

where *x* is the spatial location, and *t* is time. APAR_(*x*,__*t*)_ and *ε*_(*x*,__*t*)_ are calculated using Eqs. (4) and (5), respectively.

(4) $$APAR_{\left(x,t\right)}=SOL_{\left(x,t\right)}\times FPAR_{\left(x,t\right)}\times 0.5$$

where SOL_(*x*,__*t*)_ is the total solar radiation (MJ m^−2^) of pixel *x* at time *t*, and FPAR_(*x*,__*t*)_ is the fraction of the photosynthetically active radiation absorbed by vegetation. FPAR_(*x*,__*t*)_ can be determined from NDVI data (Zhu et al., 2005); 0.5 represents the proportion of the total solar radiation available for vegetation.

The actual light use efficiency is the efficiency with which the energy absorbed by vegetation is transformed into carbon (C), as a proportion of dry organic matter, through fixation of solar radiation and photosynthesis (Potter & Klooster, 1997) and is mainly influenced by temperature and moisture (Field, Randerson & Malmström, 1995).

(5) $$\varepsilon_{\left(x,t\right)}=T_{\varepsilon 1\left(x,t\right)}\times T_{\varepsilon 2\left(x,t\right)}\times W_{\varepsilon \left(x,t\right)}\times \varepsilon_{max}$$

where *T*_*ε*1(*x*,__*t*)_ and *T*_*ε*2(*x*,__*t*)_ denote the temperature stress coefficients of light use efficiency, *W*_*ε*(*x*,__*t*)_ is the water stress coefficient that indicates the reduction in light use efficiency caused by moisture, and *ε*_*max*_ denotes the maximum light use efficiency under ideal conditions set as the maximum possible light energy conversion efficiency. Parameters for vegetation in China may differ in value from global vegetation parameters (Peng, Guo & Wang, 2000); hence, we used the parameters for grassland maximum light use efficiency in China as simulated by Zhu et al. (2005), and this term was set uniformly at 0.542 g C MJ^−1^ for grassland in Xinjiang. A more detailed description of the algorithm for calculating and refining *W*_*ε*(*x*,__*t*)_ can be found in Zhu et al. (2005).

The CASA model was used to simulate grassland NPP in Xinjiang, and its accuracy was high (*R*^2^ = 0.69, *P* < 0.01). Therefore, the CASA model is suitable for estimating the grassland NPP of Xinjiang (Zhang et al., 2018).

### Spatial variation and correlation analysis of grassland NPP and phenology

Theil-Sen median trend analysis was used to analyze trends of grassland phenology and NPP (Burn & Elnur, 2002). The Theil-Sen median trend analysis method can reduce the effects of data outliers and is considered a reliable nonparametric statistical trend calculation method (Theil, 1950; Sen, 1968).

Pearson correlation coefficients and significance tests were used to examine the relationship between NPP and phenological shifts over 2001–2014. Two-tailed *t*-tests used to test the significance of the correlations, with *P* values < 0.05 indicating significance.

The partial correlations of grassland NPP and phenology with temperature and precipitation were analyzed.

Xinjiang experiences a continental monsoon climate, in which there is a certain correlation between precipitation and temperature. The effects of interactions between temperature and precipitation can be eliminated by calculating the coefficients of partial correlations of grassland phenology and NPP with temperature and precipitation. Thus, partial correlation coefficients are used in all the correlation analyses in this study.

Research has shown that the effects of climate on vegetation are lagged and cumulative (Shen et al., 2014). Therefore, for the analyses of vegetation phenology and climatic factors, two climatic factors, average temperature and cumulative precipitation, were selected for the 6 months prior to the grassland vegetation SOS (i.e., from May to December) and the 6 months before the EOS for grassland vegetation (i.e., October to May). Then, the relationships between grassland vegetation phenology and temperature and precipitation in Xinjiang over the past 14 years were analyzed. Partial correlation analyses of annual temperature and precipitation with annual grassland vegetation NPP were performed.

## Results

### Spatial distribution and variation of grassland NPP and phenological metrics

**Figure 2:**
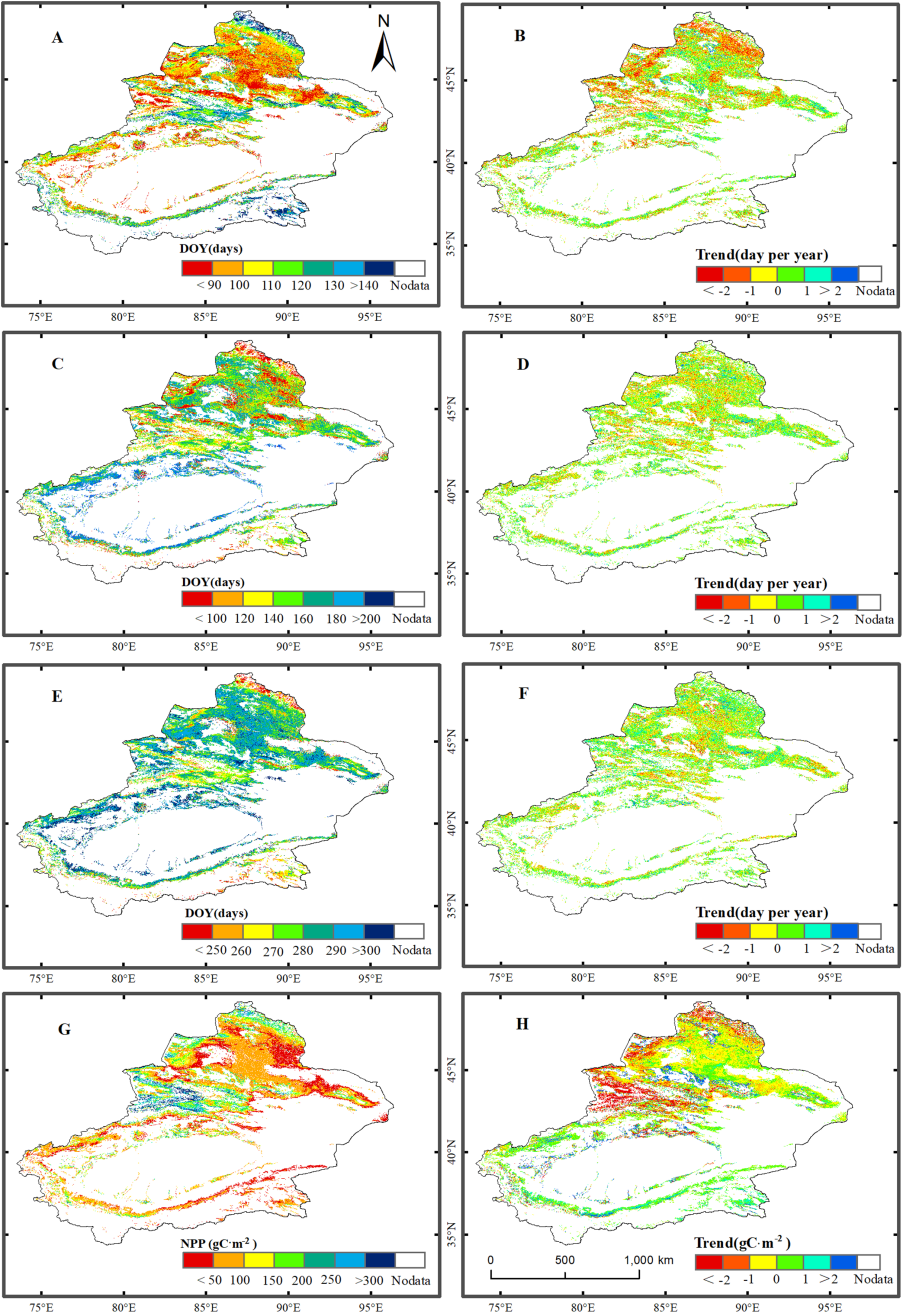
Trend analysis and spatial distribution of multiyear average values for the phenological metrics of grassland in Xinjiang for the period 2001–2014. (A, C, E, & G) The spatial distributions of multiyear average values for the SOS, EOS, LOS, and NPP, respectively; (B, D, F, & H) spatial trends in SOS, EOS, LOS, and NPP, respectively.

Figure 2 shows the spatial distributions of the average values of the phenological metrics (SOS, EOS, and LOS) and NPP of grassland in Xinjiang from 2001–2014. As illustrated in Fig. 2, there were notable regional differences in phenological metrics and NPP in Xinjiang. The SOS of the grassland in the low-mountain regions occurred the earliest, followed by that in the high-mountain zones. The areas for which the SOS occurred between the 90th and 140th days accounted for 72.5% of the total grassland area. The earliest SOS dates occurred primarily in the Junggar Basin, Ili River Valley, and Tarim Basin. The season started after the 140th day in certain regions at relatively high altitudes, such as the Altai, Tianshan, and Kunlun Mountains. The EOS occurred relatively early in northern Xinjiang. The EOS in most of the northern regions of Xinjiang occurred before the 290th day. The EOS in southern Xinjiang occurred relatively late and varied with altitude. The areas for which the EOS occurred between the 250th and 300th days accounted for 86.1% of the total grassland area. The earliest EOS occurred in the Altai Mountains and in certain regions of the Junggar Basin. The LOS varied significantly with altitude. The LOS was relatively long in the Altai, Tianshan, and Kunlun Mountains, and the LOS at the periphery of the Tarim Basin was relatively short. The areas for which the LOS was 100–200 d accounted for 82.8% of the total grassland area. The average NPP of the grassland over all of Xinjiang was 113.5 g C/m^2^. Among the various regions in Xinjiang, the NPP of the grassland in the relatively high-altitude regions of the Ili River Valley and the Altai Mountains was the highest, followed by that of the midmountain regions of the Tianshan and Altai Mountains. The NPP was lowest in certain regions of the Tarim and Junggar Basins.

Figure 2 shows the trends for phenological metrics and NPP of grassland in Xinjiang for 2001–2014. In Xinjiang, the proportion of grassland area with an advanced SOS (55.2%) was greater than that of grassland area with a delayed SOS (44.8%). Additionally, the SOS varied over a range of ±1 d, accounting for 63.8% of the total grassland area. The SOS was advanced primarily in the midmountain regions of the Altai Mountains and the Ili River Valley. Grassland for which the SOS was delayed was distributed sporadically at the margin of the Junggar Basin and on the northern slopes of the Tianshan Mountains. The proportion of grassland area for which the EOS was advanced (49.1%) was slightly lower than that of grassland area with delayed EOS (50.9%). Grassland area for which the EOS varied over a range of ±1 d accounted for 67.4% of the total grassland area. Grassland area for which the EOS was advanced was distributed sporadically in the midmountain region of the Ili River Valley and the Junggar Basin. In Xinjiang, the proportion of grassland area with an increased LOS (54.6%) was slightly larger than that of grassland area with a decreased LOS (45.4%). Grassland area for which the LOS varied over a range of ±1 d accounted for 66.8% of the total study area. There was an overall increase in the NPP of the grassland in the study area. In addition, the NPP of 61.6% of the grassland area, which was primarily distributed in the Junggar and Tarim Basins, increased, while the NPP of 38.4% of the grassland area, which was primarily distributed across the Ili River Valley and in the Altai Mountains, decreased.

### Correlation analysis of grassland NPP and phenological metrics

**Figure 3:**
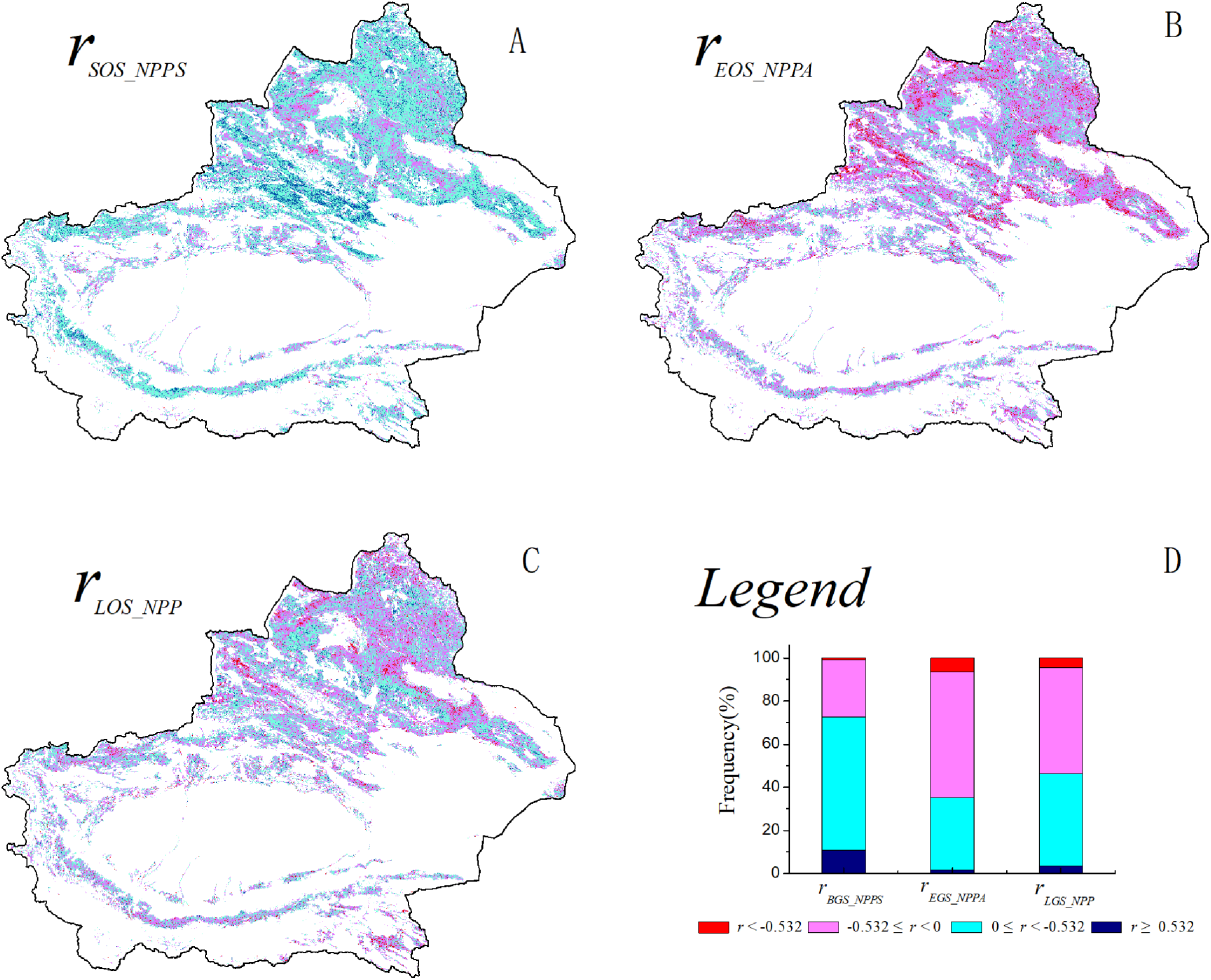
Spatial distribution of correlation coefficients between grassland NPP and phenological metrics. (A) The spatial distribution of the correlation between the grassland SOS and spring NPP; (B) the spatial distribution of the correlation between the grassland EOS and autumn NPP; (C) the spatial distribution of the correlation between the grassland LOS and NPP; (D) illustrations of ABC.

The spatial correlation between the SOS and spring NPP was analyzed for the grassland in Xinjiang from 2001–2014 (Fig. 3A). A negative correlation was found between the SOS and spring NPP in 72.7% of the total grassland area. A significant negative correlation was found between the SOS and spring NPP in 10.9% of the grassland area (*P* < 0.05), which was primarily distributed along the southern slopes of the Tianshan Mountains, in the Ili River Valley, and in certain regions of the Kunlun Mountains. A positive correlation was found between the SOS and spring NPP in 27.5% of the grassland area. A significant positive correlation was found between the SOS and spring NPP in a very small proportion of the grassland area. A positive correlation was found between the EOS and autumn NPP for a large proportion (64.7%) of the grassland area (Fig. 3B). A significant positive correlation was found between the EOS and autumn NPP in 6.3% of the grassland area (*P* < 0.05), which was primarily distributed in certain regions of the southern and northern slopes of the Tianshan Mountains. There were no significant differences between the proportions of grassland area for which the LOS and NPP were positively correlated (53.3%) and the proportions of grassland area for which the LOS and NPP were negatively correlated (46.7%) (Fig. 3C). Spatially, there were no notable regions in which there was a positive or negative correlation between the LOS and NPP.

**Figure 4:**
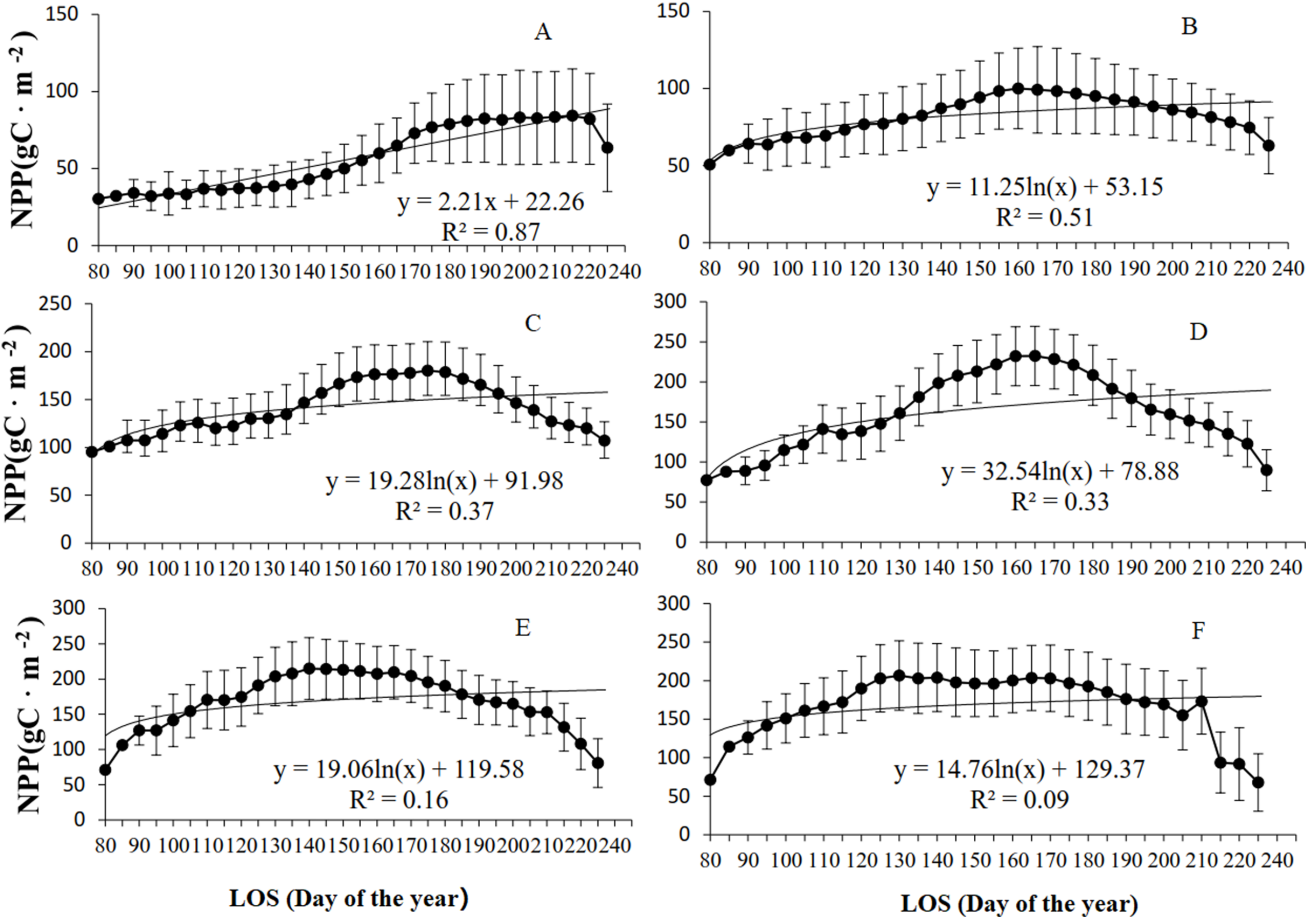
Relationship between grassland NPP and LOS at different *K* levels in Xinjiang from 2001–2014. (A, B, C, D, E, & F) The relationships between NPP and LOS from 2001–2014 in extra-arid, arid, semiarid, subhumid, humid, and perhumid regions, respectively.

The relationship between grassland NPP and LOS from 2001–2014 was further analyzed at various *K* levels (Fig. 4). As shown in Fig. 4, grassland NPP initially increased but then decreased with increasing LOS at different levels of *K*. Of the four (linear, logarithmic, exponential, and power) models established for grassland LOS and NPP, the exponential model exhibited the highest *R*^2^ for all the regions, except for in arid regions (determined based on the *K* level), for which the linear model showed the highest *R*^2^. For example, in arid regions, the maximum NPP of the grassland (100.1 g C/m^2^) occurred for a LOS of 160 d. When the LOS was shorter than 160 d, the following relationship was found between NPP and LOS of the grassland: slope = 2.74, *R*^2^ = 0.97, and *P* < 0.01. When the LOS was longer than 160 d, the following relation was found between the grassland NPP and LOS: slope = −2.45, *R*^2^ = 0.91, and *P* < 0.01. As the *K* level changed from semiarid to subhumid to humid to perhumid, there was a decrease in the grassland LOS when NPP reached the maximum level. For example, in semiarid regions, the maximum grassland NPP (180.2 g C/m^2^) occurred for a LOS of 175 d. In subhumid regions, the maximum NPP (232.4 g C/m^2^) occurred at a LOS of 165 d. In humid regions, the maximum NPP (213.9 g C/m^2^) occurred with a LOS of 140 d. In perhumid regions, the maximum grassland NPP (203.9 g C/m^2^) occurred for the shortest LOS of 130 d. There were no strong correlations between grassland NPP and LOS in regions across various *K* levels, except for in grassland in extra-arid regions. However, the piecewise function showing an increase or decrease in NPP demonstrated a significant correlation between grassland LOS and NPP. Moreover, as the *K* level changed from extra-arid to perhumid, there was an increase in NPP but a decrease in the LOS.

**Figure 5:**
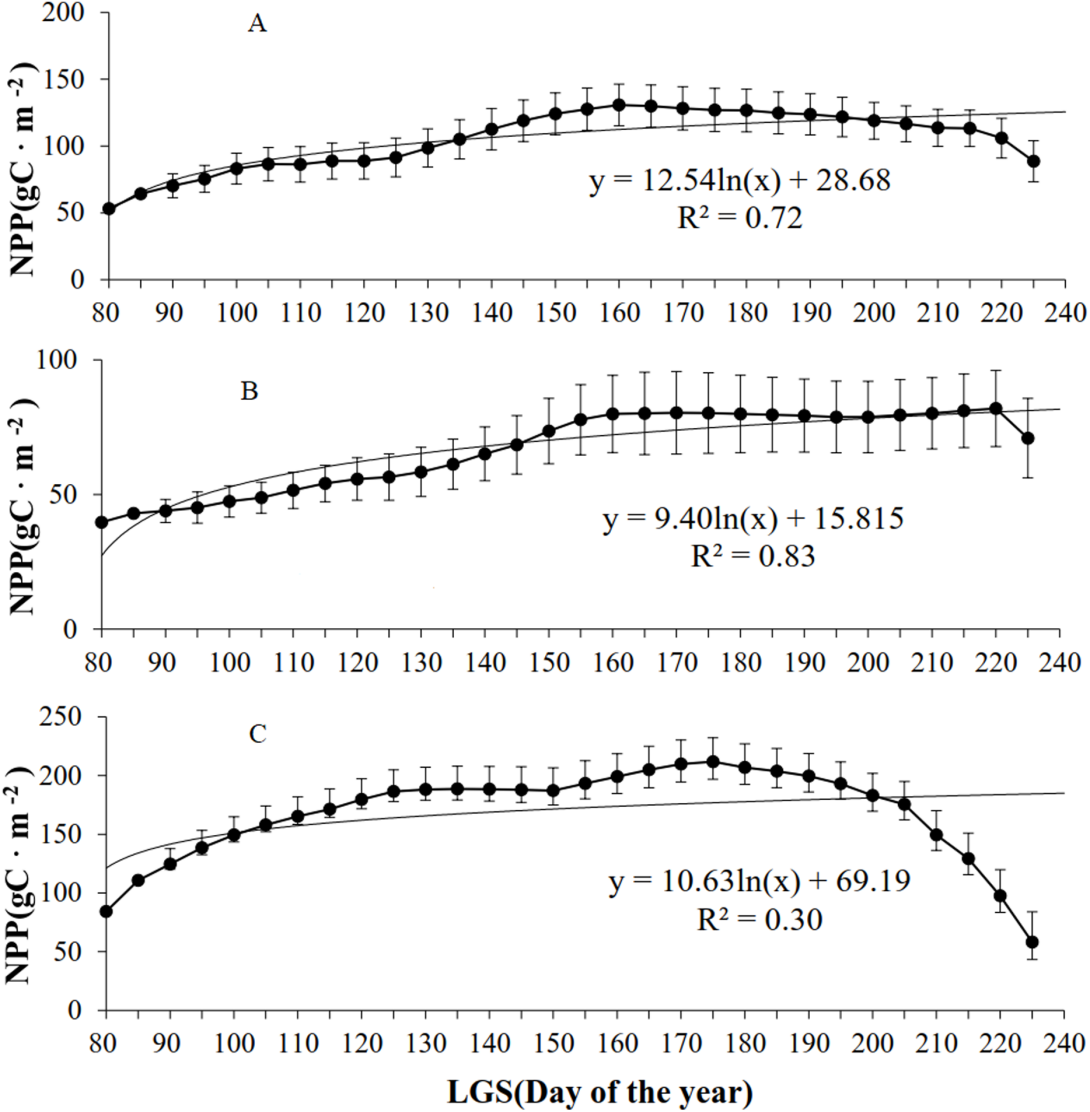
Relationships between NPP and LOS of grassland types in Xinjiang from 2001–2014. (A, B, & C) The relationships between NPP and LOS from 2001–2014 in steppe, desert grassland, and meadow, respectively.

According to the analysis of grassland types (Fig. 5), the NPP of all the grassland types initially increased but then declined with increasing LOS. Of the four (linear, logarithmic, exponential, and power) models established for the LOS and NPP, the exponential model exhibited the highest *R*^2^ values for the three grassland types. For the steppe grassland type, the maximum NPP (130.7 g C/m^2^) occurred at a LOS of 160 d, with *R*^2^ = 0.72. For the desert grassland type, the maximum NPP (81.9 g C/m^2^) occurred for a LOS of 220 d, with *R*^2^ = 0.86. For the meadow grassland type, the maximum NPP (211.8 g C/m^2^) occurred with a LOS of 175 d, and *R*^2^ = 0.35.

### Characteristics of grassland NPP and phenological metrics under various climatic conditions

**Figure 6:**
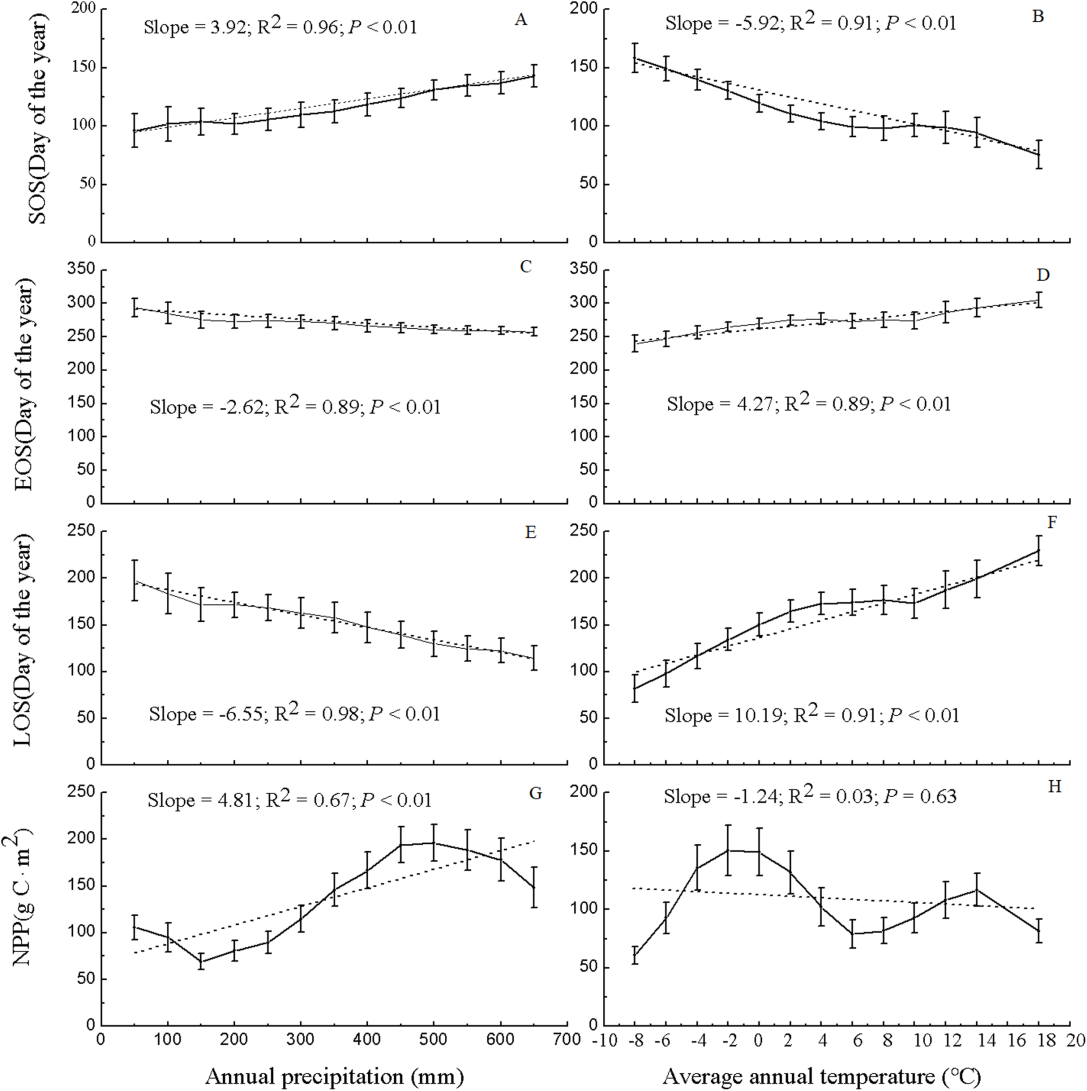
Variation in NPP and phenological data for grassland in Xinjiang under various precipitation and temperature conditions from 2001–2014. (A, C, E, & G) Differences in NPP and phenological data for grassland in Xinjiang under various precipitation conditions from 2001–2014 in terms of SOS, EOS, LOS, and NPP, respectively; (B, D, F, & H) variations in NPP and phenological data for grassland in Xinjiang under various temperature conditions from 2001–2014 in terms of SOS, EOS, LOS, and NPP, respectively.

Figure 6 shows the values of the phenological metrics and NPP of grassland in regions with various temperature and precipitation conditions. In the majority of the vegetated regions of Xinjiang, the temperature varies between −8 and 18 °C, and the precipitation varies between 50 and 650 mm. Thus, based on the temperature and precipitation ranges in the majority of vegetated regions, the values of the phenological metrics and NPP of grassland in Xinjiang were statistically analyzed at temperature intervals of 2 °C and precipitation intervals of 50 mm. Along various temperature and precipitation gradients, the relationships between grassland phenological metrics and NPP varied significantly. As precipitation increased, the SOS was notably delayed (slope = 3.92, *R*^2^ = 0.96, and *P* < 0.01), the EOS advanced significantly (slope = −2.62, *R*^2^ = 0.89, and *P* < 0.01), and the LOS decreased significantly (slope = −6.55, *R*^2^ = 0.98, and *P* < 0.01). The pattern for grassland NPP was relatively complex with increasing precipitation. Specifically, as the precipitation increased, the NPP of the grassland at first decreased, then increased, and then decreased again; overall, grassland NPP increased in a fluctuating pattern (slope = 4.81, *R*^2^ = 0.67, and *P* < 0.01). With 500 mm of precipitation, the maximum NPP (196.18 g C/m^2^) occurred. As the temperature increased, the SOS for grassland advanced significantly (slope = −5.92, *R*^2^ = 0.91, and *P* < 0.01), the EOS was delayed significantly (slope = 4.27, *R*^2^ = 0.89, and *P* < 0.01), and the LOS increased significantly (slope = 10.19, *R*^2^ = 0.91, and *P* < 0.01). Additionally, with increasing temperature, grassland NPP initially increased, then decreased, and then increased again; overall, the NPP decreased (slope = −1.24, *R*^2^ = 0.03, and *P* = 0.63), albeit nonsignificantly. At a temperature of −2 °C, the maximum NPP occurred (150.25 g C/m^2^).

Table 1 summarizes the partial correlations of temperature and precipitation with the grassland SOS, EOS, and NPP. For each of the three grassland types, the area with a negative partial correlation between the grassland SOS and the 6-month (December through May) average temperature is significantly greater than the area with a positive partial correlation between these factors, whereas the area with a negative partial correlation between the grassland SOS and the 6-month (December through May) average precipitation is significantly smaller than the area with a positive partial correlation between these factors. These findings suggest that higher temperature in December through May in Xinjiang favors an earlier grassland SOS, whereas higher precipitation in December through May in Xinjiang is unfavorable to earlier grassland SOS. For each of the three grassland types, the area with a positive correlation between grassland EOS and the 6-month (May through October) average temperature is slightly greater than the area with a negative correlation between these factors, whereas the area with a negative correlation between the grassland EOS and the 6-month (May through October) average precipitation is slightly greater than the area with a positive correlation between these factors. These findings suggest that temperature in May through October can advance the grassland EOS, whereas precipitation in May through October can delay the grassland EOS. Increased annual precipitation and temperature can both increase grassland NPP in Xinjiang.

**Table 1:** Analysis of partial correlations of grassland phenological parameters and NPP with temperature and precipitation.

Tapes	Desert grassland (%)		Steppe grassland (%)		Meadow grassland (%)	
	*r* < 0	*r* > 0	*r* < 0	*r* > 0	*r* < 0	*r* > 0
SOS-Temperature	71.09	28.91	77.85	22.15	77.64	22.36
SOS-Precipitation	32.04	67.96	25.56	74.44	27.31	72.69
EOS-Temperature	47.71	52.29	43.31	56.69	45.73	54.27
EOS-Precipitation	50.91	49.09	57.48	42.52	53.34	46.66
NPP-Temperature	42.27	57.73	34.83	65.17	30.74	69.26
NPP-Precipitation	22.03	77.97	23.40	76.60	49.14	50.86

## Discussion

### Spatial distributions of grassland phenological metrics and NPP

As illustrated in Fig. 2, grassland in the relatively warm regions (e.g., the Junggar and Tarim Basins) featured a relatively early SOS, a relatively late EOS, and a relatively long LOS. In comparison, grassland in the relatively cool regions (e.g., the Tianshan, Altai, and Kunlun Mountains) exhibited a late SOS, an early EOS, and a relatively short LOS. The regions with relatively high precipitation and lower temperature were located at relatively high altitudes (e.g., the Tianshan, Altai, and Kunlun Mountains). The grassland in these regions showed a relatively late SOS, a relatively early EOS, and a relatively short LOS. Conversely, grassland in regions with relatively low precipitation had a relatively early SOS, a relatively late EOS, and a relatively long LOS. Grassland with a relatively early EOS was primarily found in the Altai Mountains, which are located at relatively high altitudes (Fig. 1). This region has comparatively low temperatures and relatively high precipitation. The EOS of the vegetation in this region was notably earlier than that in other grassland regions. As shown in Table 1, higher average temperature in the first 6 months advanced the grassland SOS in most regions of Xinjiang, whereas higher precipitation delayed the grassland SOS in most regions. Higher average temperature during the first 6 months delayed the EOS of most grassland regions, whereas greater precipitation advanced the grassland EOS in most regions. However, grassland in certain regions of the Junggar Basin unexpectedly also experienced a relatively early EOS. This finding may be related to the local grassland type. The vegetation in the Junggar Basin is relatively sparse. After a period of growth, the leaves of the vegetation in the Junggar Basin are yellowish, long, and thin. Additionally, the vegetation cover in the Junggar Basin is often low. As a result, during the information extraction process, the target signals can be easily “polluted” by background noise (primarily soil) (Townshend & Justice, 1986). The vegetation phenology in different grassland types is influenced by genetics, although the environment plays a role in regulating phenological changes (Yu et al., 2003; Zhang et al., 2019). Vegetation with a relatively high NPP was distributed primarily in the Ili River Valley and on the Altai Mountains. The grassland NPP in the Junggar and Tarim Basins was relatively low. The Junggar Basin and the peripheral region of the Tarim Basin have relatively low precipitation but extremely high evaporation rates. By contrast, the Ili River Valley and the Altai Mountains have relatively abundant precipitation and relatively low evaporation rates and are therefore suitable for vegetation growth. Spatially, grassland NPP was relatively low in regions in which the LOS was relatively long. For example, the LOS of grassland at the periphery of the Tarim Basin as well as the Junggar Basin was relatively long, but the NPP of the grassland was relatively low. In comparison, the LOS of grassland in the Ili River Valley and in the Altai Mountains was relatively short, but the NPP of the grassland was relatively high. The NPP in Xinjiang was high from June–August. The highest grassland NPP occurred in July.

### Trends in the grassland phenological metrics and NPP

Extensive research has shown that the SOS of vegetation at high latitudes in the Northern Hemisphere has advanced (Zhang et al., 2013; Jeganathan, Dash & Atkinson, 2014). Our study also shows that the SOS of grassland in most regions of Xinjiang has advanced. In particular, the SOS of grassland in certain high-altitude regions (e.g., the Altai and Tianshan Mountains) has advanced significantly. In these regions, because there is an abundant water supply, temperature is the primary factor that limits grassland growth. As the overall climate in Xinjiang has become warmer and wetter in recent years (Fang et al., 2013), the temperature increases will correspondingly cause the SOS to advance, particularly in the Altai and Tianshan Mountains, two regions with abundant precipitation but relatively low temperatures. During this study, the proportion of grassland for which the EOS was delayed was found to be slightly greater than that for grassland with an advanced EOS. This finding is consistent with that of Menzel et al. (2006), who reported an advance in SOS in their study system. However, they found no notable advancement of or delay in the EOS. The EOS is affected by a multitude of factors such as temperature, precipitation, and available nutrients. Therefore, the EOS for vegetation is not delayed by temperature increases (Piao et al., 2008). In the present study, the proportion of vegetation for which the overall LOS was elevated was found to be greater than that for vegetation with an overall decrease in LOS. This finding is consistent with the result obtained by Zeng, Jia & Epstein (2011) using MODIS data, which indicated an increase in the LOS of vegetation in the Northern Hemisphere. However, there was no distinct distribution pattern for the regions in which the LOS increased or decreased. There was a decrease in the NPP of grassland in the Ili River Valley and in the Altai Mountains, two high-altitude regions. This finding further demonstrates that advancement of the grassland SOS does not play a decisive role in annual NPP. However, there was an increase in NPP of grassland in the majority of the other Xinjiang regions. This observation indicates that the climate in Xinjiang is becoming increasingly suitable for grassland growth. Evidently, the variation in grassland NPP is also, to a certain extent, related to other factors such as human activity (Zhang et al., 2018). Overall, as the climate in Xinjiang becomes warmer and wetter (Fang et al., 2013), there will be an increase in both the LOS and NPP of the grassland in Xinjiang.

### Relations between the grassland phenological metrics and NPP

Chen et al. (2016) studied the relationship between the spring NPP and SOS of vegetation in the Northern Hemisphere using Community Land Model 4.5, and they found that the earlier the SOS arrived, the higher the spring NPP was. The present study revealed a general increase in NPP in Xinjiang in March and April. Because the SOS in Xinjiang occurs largely in March and April (Zhang et al., 2019), a negative correlation was found between the SOS and spring NPP in most of the regions in this study. This result suggests that advancement of the SOS can help increase spring NPP. A positive correlation was found between autumn NPP and SOS over a large area of Xinjiang. This finding suggests that a delay in the EOS will lead to an increase in autumn NPP. From 2001–2014, there was no significant delay in or advancement of the grassland EOS in Xinjiang. NPP decreased in September but increased in October and November. Therefore, overall, there was no notable increase or decrease in autumn grassland NPP.

In August and September, there was a decrease in the NPP of the grassland in most Xinjiang regions. As a result of the elevated temperature, there was a decrease in the amount of water in the soil that could be used by plants. Advancement of the SOS requires relatively high consumption of soil water. This increase may result in a decrease in the amount of water that can be used by plants in August and September (Wolf et al., 2016). An increase in temperature will notably increase the vegetation LOS. In this study, the correlation between grassland NPP and vegetative respiration was not explicitly examined. However, many studies have demonstrated that temperature affects metabolic processes (e.g., photosynthesis, respiration, and, to some extent, organic matter synthesis and transport) of vegetation and that an increase in temperature will lead to an increase in the consumption of organic matter by plants (Piao et al., 2008; Dragoni et al., 2011). For example, Piao et al. (2008) found that higher temperatures lead to a longer LOS and higher NPP and photosynthetic rate but also enhance respiration and may thereby cause a decrease in NPP. Dragoni et al. (2011) found that the consumption of NPP by respiration resulting from a long growing season was not greater than the increase in NPP resulting from photosynthesis and that there was an increase in NPP during long growing seasons. Evidently, effects of environmental changes on respiration and assimilation differ significantly among various ecosystems (Falge et al., 2002). Piao et al. (2008) primarily studied evergreen forests. Dragoni et al. (2011) focused largely on broadleaved deciduous forests. The present study centered mostly on grassland regions and examined only a small number of forest regions. Additionally, the impacts of changes in the growing season on NPP are related to the structure, age, geographical spatial distribution, and rhizospheric microorganisms of the vegetation (Phillips, Bernhardt & Schlesinger, 2009; Dragoni et al., 2011; Girardin et al., 2016). Regardless of the vegetation type, there is a close relationship between the LOS and NPP of vegetation.

Figure 6 shows that at various *K* levels, grassland NPP initially increased but then decreased with increasing LOS. This finding is inconsistent with that of Wang et al. (2017) who found that at temperatures from −6 °C to 2 °C, NPP initially increased but then decreased with increasing LOS. Additionally, Wang et al. (2017) found that NPP initially decreased but then increased with increasing LOS at precipitation levels higher than 600 mm but did not find similar trends for other temperature and precipitation ranges. The differences between our findings and those of Wang et al. (2017) may have occurred because we considered the combined action of temperature and precipitation, whereas Wang et al. (2017) considered the effects of temperature or precipitation alone on the response of NPP to the LOS. This association between NPP and LOS is significantly related to photosynthesis and respiration (Piao et al., 2008; Chen et al., 2016). In the initial stage of the growing season, the temperature is relatively low and photosynthesis is stronger than respiration. As the LOS increases, the temperature becomes relatively high, and respiration increases slowly. As respiration increases to the same level as photosynthesis, the NPP of the vegetation reaches its maximum. The present study found that overall, there was an increase in NPP in regions that spanned various *K* levels, except in the perhumid regions. This observation suggests that during years with a relatively long growing season, relatively high early-spring NPP offsets or exceeds the NPP consumed in the summer, when respiration increases (Richardson et al., 2010; Chen et al., 2016). Additionally, the relationship between the NPP and LOS of the vegetation was found to vary with the *K* level. The lower the *K* level was, the longer the LOS was when NPP reached the maximum level. The higher the *K* level was, the shorter the LOS was when NPP reached the maximum. In this study, the NPP of the vegetation in Xinjiang was relatively low in regions where the LOS was relatively long (e.g., in the extra-arid, arid, and semiarid regions). In contrast, the NPP was relatively high in regions where the LOS was relatively short (e.g., the subhumid, humid, and perhumid regions).

### Effects of climatic factors on grassland NPP and phenological metrics

**Figure 7:**
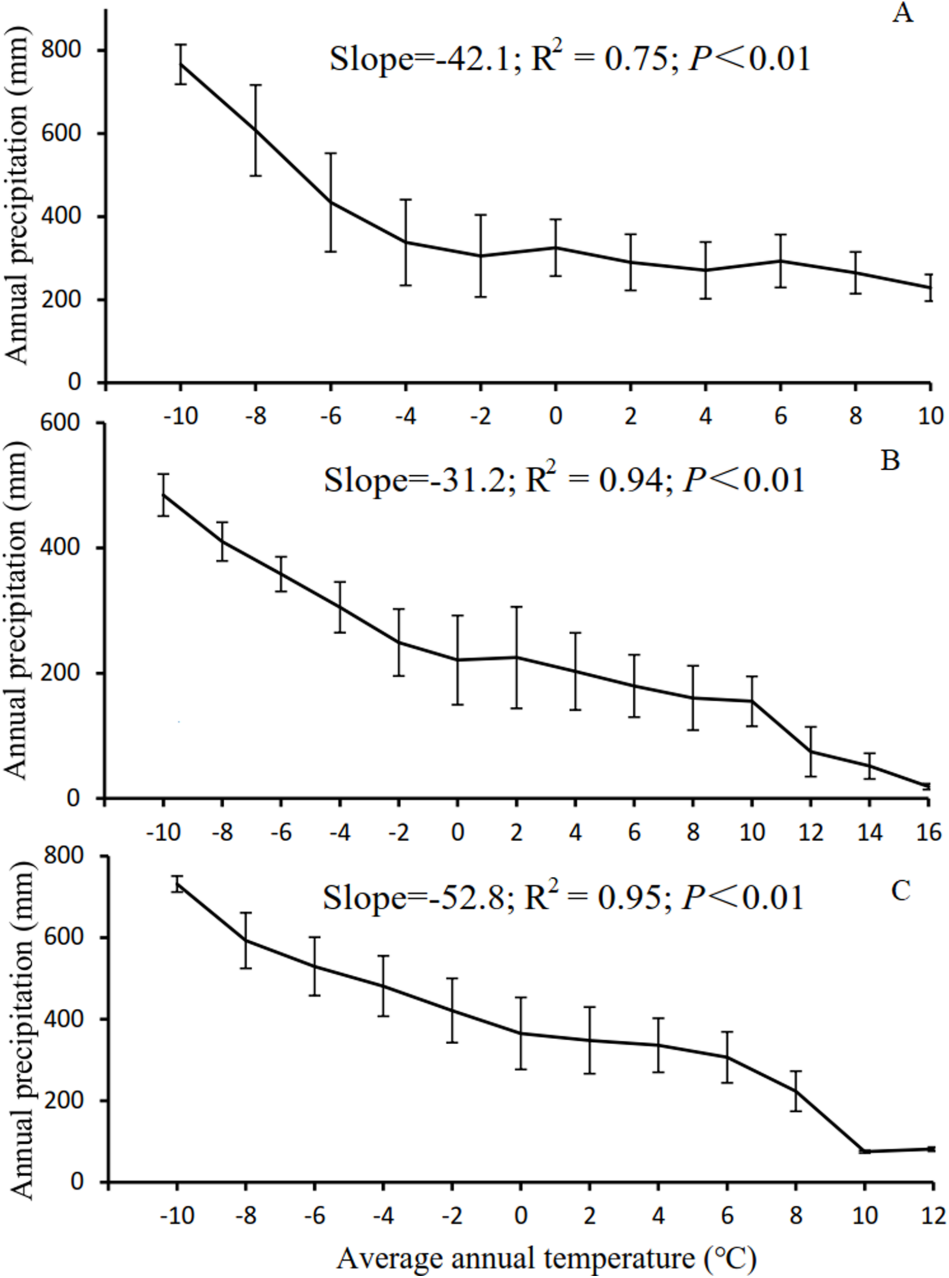
Correlation between temperature and precipitation in Xinjiang. (A, B, & C) Correlations between temperature and precipitation in steppe, desert grassland, and meadow, respectively.

The results in Section 3.3 show that as the temperature and precipitation increased, there was a notable advancement of or delay in the phenological period. However, this phenomenon was not observed for NPP. As the temperature increased from −8 to 18 °C, the SOS advanced significantly, whereas the EOS was delayed significantly. As the precipitation increased from 50 to 650 mm, the SOS of the vegetation was delayed significantly, whereas the EOS advanced significantly. As illustrated in Fig. 7, the low-temperature regions of Xinjiang experience high precipitation in three main grassland types. These regions are generally distributed in the high-altitude Altai, Kunlun, and Tianshan Mountains. In these regions, the SOS for grassland occurred relatively late, whereas the EOS occurred relatively early, as shown in Fig. 2. High-temperature regions have relatively low precipitation and low *K*. These regions are generally located in the low-altitude Junggar and Tarim Basins. Here, the SOS occurred relatively early, whereas the EOS occurred relatively late. However, the variation in NPP in these regions was relatively complex (Fig. 6). With increasing temperature, NPP first increased, then decreased, then increased again, and finally decreased again. Overall, NPP decreased as temperature increased. The maximum NPP (150.25 g C/m^2^) occurred at a temperature of −2 °C. With increasing precipitation, NPP first decreased, then increased, and then decreased again. Overall, the grassland NPP increased as precipitation increased. The maximum NPP (196.18 g C/m^2^) occurred with a precipitation level of 500 mm. Notably, the maximum NPP did not occur in regions with the highest temperature or precipitation. This observation suggests that, in addition to the combined action of temperature and precipitation, NPP is also affected by other factors such as human interference (Zhang et al., 2018), day length (Kaduk & Heimann, 1996), soil moisture content (Vitasse & Basler, 2013), and slope gradient (Liang et al., 2016). Evidently, with increases or decreases in temperature and precipitation, the phenological period linearly increased or decreased. However, the variation in NPP was relatively complex. This trend further validates the results shown in Fig. 6, in which NPP increased or decreased only when the LOS increased or decreased to below a certain threshold.

## Conclusions

This study primarily examined the spatial patterns and trends in grassland phenological metrics and NPP in Xinjiang, the values of the phenological metrics and NPP in Xinjiang regions differing in temperature and precipitation conditions, and the relations between grassland NPP and phenological metrics. From 2001–2014, the proportion of grassland area for which the SOS was advanced was greater than that with delayed SOS. The proportion of grassland area with a delayed EOS was slightly greater than that of grassland with an advanced EOS. There was an increase in the LOS in a large number of regions. There was an increase in the NPP of grassland in regions at low altitudes, such as the Junggar Basin and the periphery of the Tarim Basin. In contrast, there was a decrease in the NPP of grassland in regions at high altitudes, such as the Ili River Valley and the Altai Mountains. The proportion of grassland area with an increased NPP was greater than that of grassland with a decreased NPP. The proportion of grassland area with an increased NPP was greater than that of grassland with a decreased NPP in each month, except for in May, August, and September, when the proportion of grassland area with a decreased NPP was greater than that of grassland with an increased NPP. The highest NPP occurred from June through August. The maximum NPP occurred in July. Spatially, the earlier the SOS in a region was, the higher the spring NPP in that region was. The earlier the EOS in a region was, the lower the autumn NPP in that region was. There was no clear spatial correlation between the LOS and annual NPP. However, further linear analysis of the relation between the LOS and NPP of various types of grassland indicated that the NPP initially increased but then decreased with increasing LOS. As the *K* level changed from extra-arid to perhumid, the LOS gradually decreased when the NPP reached the maximum level. Warmer regions featured an earlier SOS, a later EOS, and thus a longer LOS. Regions with higher precipitation exhibited a later SOS, and regions with an earlier EOS experienced a shorter LOS.

## Additional Information and Declarations

### Data Availability

The following information was supplied regarding data availability: The meteorological data were obtained from the China meteorological science data-sharing service system (http://data.cma.cn/data/cdcdetail/dataCode/SURF_CLI_CHN_MUL_MON.html). In order to obtain the data, users must apply for registration on this page and state the purpose of your study. Data from all stations in Xinjiang from 2001 to 2014 were used. The main meteorological elements are average temperature and 20-20 hour precipitation. Due to copyright restrictions, this dataset can be accessed after completing registration for a Username and Password. MODIS vegetation phenological (MCD12Q2) data, which have a spatial resolution of 500 m for the period 2001-2014, are available at NASA: https://ladsweb.modaps.eosdis.nasa.gov/search/order/1/MCD12Q2.
